# Application of a mouse model humanized for cytochrome P450–mediated drug metabolism to predict drug-drug interactions between a peptide and small molecule drugs

**DOI:** 10.1016/j.dmd.2025.100153

**Published:** 2025-09-02

**Authors:** Yury Kapelyukh, Charlotte Gabel-Jensen, Alastaire Kenneth MacLeod, Kevin-Sebastien Daniel Coquelin, Laste Stojanovski, Laura Frame, Amy Tavendale, Colin J. Henderson, Kevin D. Read, Charles Roland Wolf, Carolina Säll

**Affiliations:** 1 Cancer Research Division, University of Dundee Medical School, Dundee, United Kingdom; 2 Development ADME, Novo Nordisk A/S, Måløv, Denmark; 3 Drug Discovery Unit, Wellcome Centre for Anti-Infectives Research, Division of Biological Chemistry, University of Dundee, Dundee, United Kingdom; 4 Department of Molecular Biology and Genetics, Aarhus University, Aarhus, Denmark

**Keywords:** Drug/drug interactions, Humanized models, Cytochrome P450, Peptide/small molecule drug-drug interaction, Drug-drug interaction predictions, Glucagon-like peptide 1

## Abstract

Conventional preclinical in vitro approaches inaccurately predicted clinical trial outcomes of drug-drug interactions involving the peptide NN1177, a glucagon and glucagon-like peptide 1 receptor coagonist. To further study the mechanisms behind this discrepancy, we have exploited a mouse model (8HUM) humanized for the major cytochrome P450 (P450) enzymes involved in drug disposition in humans. We show that NN1177 administration to 8HUM mice suppressed hepatic in vivo expression of CYP3A4 (82% compared to vehicle) and CYP1A2 (58% compared to vehicle). This was consistent with in vitro sandwich culture hepatocyte data reported previously. However, reduction in CYP3A4 and CYP1A2 levels in vivo appeared to resolve over time, despite daily NN1177 administration. These findings suggest an adaptive response to the metabolic effects of NN1177. In vivo pharmacokinetic studies in 8HUM closely matched the findings observed in the clinical trial, because there was no relevant increase in the exposure of the CYP3A4 and CYP1A2 probe drugs. Furthermore, no suppression effects were observed when the mice had been pretreated with the inducing agents, St. John’s wort or phenobarbital, respectively, suggesting that the mechanism of P450 reduction does not involve the transcription factors constitutive androgen receptor or pregnane X receptor. These data highlight the complexities associated with therapeutic peptide drug-drug interactions and the remaining challenges for accurate predictions of P450 suppression and potential clinical implications. The humanized 8HUM model provides a promising and informative preclinical tool that can add high value during drug development by providing further insights into the effects on P450 expression, together with the subsequent impact of coadministered probe drugs in an in vivo model.

**Significance Statement:**

The current work describes the application of a humanized cytochrome P450 mouse model that provides further insight into the potential mechanisms and outperforms conventional in vitro approaches for preclinical predictions of peptide drug-drug interaction risk. The results showed no significant effects on the Cooperstown 5 + 1 cocktail, in line with clinical findings, and thereby represent an exciting model to further explore future therapeutic peptide projects during drug development.

## Graphical abstract

**Figure:**
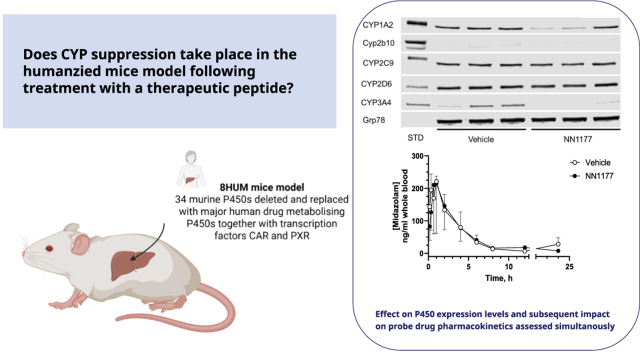

## 1 Introduction

The use of therapeutic peptides has gained significant attention due to their potential to treat a wide range of medical conditions. As a drug modality, peptides are placed in a gray zone between small molecules and large biologics such as proteins.^1^^,^^2^ The development of therapeutic peptides as novel drugs can face multiple unique challenges during the preclinical stage, because the regulatory landscape can be particularly difficult to navigate. To safeguard patients, an important part of the drug development process is to evaluate and assess the risk of clinical drug-drug interactions (DDIs). There remains a lack of detailed regulatory guidelines from health authorities^3^ on assessing the DDI potential for therapeutic peptides. This gap complicates the evaluation of how peptides may influence the expression and activity of cytochrome P450 (P450) enzymes, which are crucial for the metabolism of small-molecule comedications.

In a previous study,^4^ we demonstrated that the investigational peptide NN1177 reduced the expression of CYP3A4 and, to a lesser extent, CYP2B6 and CYP1A2 in freshly isolated human hepatocytes. This down-regulation raised concerns about potential clinical DDIs. Consequently, a clinical study employing the Cooperstown 5 + 1 drug cocktail was conducted to evaluate these interactions in the clinic. Contrary to the in vitro predictions, the pharmacokinetic (PK) analysis of the cocktail drugs revealed no significant impact on the area under the curve (AUC_0–inf_) for CYP3A4 (midazolam) or CYP2C9 (*S*-warfarin) probe drugs when coadministered with NN1177. Probe drugs for CYP2C19 (omeprazole), CYP1A2 (caffeine), and CYP2D6 (dextromethorphan) exhibited decreases in AUC_0–inf_. These unexpected results highlight a major challenge with using the current gold standard in in vitro models when assessing DDI risk for therapeutic peptides. In a follow-up study^5^ using more complex in vitro models, such as the micropatterned coculture hepatocyte system HepatoPac, NN1177 showed no down-regulation of the investigated P450 enzymes. Together, these findings further highlight the challenges associated with interpreting in vitro DDI results for peptide-based therapies and raise important questions about the necessity of subsequent clinical trials based solely on in vitro data.

The DDIs involving biological therapeutics like peptides are likely also influenced by various endogenous factors that can alter P450 enzyme expression, including inflammatory mediators (IL2, IL6, TNF, IFN), growth factors, and retinoic acid signaling.^6^, ^7^, ^8^, ^9^ Additionally, physiological factors such as gastric emptying, kidney function, and blood flow may also play significant roles.^10^ These multifaceted interactions suggest that current in vitro models may be insufficient to fully capture the net effect on P450 enzymes, underscoring the need for more complex in vivo models.

Historically, the use of animal models for preclinical DDI assessment has been hindered by significant species differences in metabolism and gene expression regulation, making extrapolation to human outcomes problematic.^11^ For instance, the human CYP1A2 reference inducer omeprazole exhibits no inductive effect in rats, and the potent human enzyme inducer rifampicin similarly shows no effect in this species.^12^^,^^13^ Such discrepancies highlight the limitations of traditional animal models in predicting human-specific enzyme induction. To address these limitations, the 8HUM mice model was developed, in which 34 murine P450s have been replaced with major human drug-metabolizing P450s along with transcription factors the constitutive androgen receptor (CAR) and the pregnane X receptor (PXR). This model has demonstrated success in predicting small-molecule DDIs and offers a promising alternative for more accurately assessing therapeutic peptide interactions.^14^, ^15^, ^16^

Because in vitro P450 down-regulation observed with NN1177 in freshly isolated human hepatocytes does not correlate with clinical DDI outcomes, we have carried out in vivo studies using the humanized 8HUM mouse. We demonstrate that this model provides a better prediction of clinical observations, thereby improving the predictive accuracy of preclinical DDI assessments for the peptide NN1177.

## 2 Material and methods

### 2.1 Chemicals

All compounds were purchased from Sigma Aldrich/Merck, with the exceptions of *S*-warfarin (Toronto Research Chemicals), midazolam (Hameln Pharmaceuticals Ltd), and St. John’s wort (SJW) (Klosterfrau Healthcare Group). All LC–MS/MS mobile phase reagents were purchased from Fisher Scientific (Thermo Fisher Scientific).

### 2.2 Animal accommodation and husbandry

All animal procedures were performed under the auspices of the Animal (Scientific Procedures) Act (1986), as amended by the European Directive 2010/63/EU, and all animal studies were approved by the Ethical Review Committee at both the University of Dundee and Novo Nordisk. Homozygous female 8HUM mice were used for experimental studies.^15^ Mice were housed on sawdust in solid-bottom, polypropylene cages and provided with RM1 pelleted diet (Special Diet Services Ltd, Stepfield) and drinking water ad libitum before and throughout the study. The temperature was maintained within the range of 19 to 23 °C and the relative humidity within the range of 40% to 70%. A 12-hour light/dark cycle was maintained. The mice were allowed to acclimatize for a minimum of 5 days before use.

### 2.3 Effect of NN1177 treatment on body weight and P450 expression in 8HUM mice

#### 2.3.1 Animal treatment

8HUM mice received single daily doses of either vehicle (s.c.; 5 mL/kg) or NN1177 (s.c.; 3 nmol/kg or 4 nmol/kg) on days 1 to 3. A dose of approximately 30 nmol/kg would be required to mimic clinical plasma exposure observed in humans. However, considering the major difference in glucagon receptor potency between mice (IC_50_ of 0.29 nM) and humans (IC_50_ of 2.54 nM), a starting dose of 3 nmol/kg was selected as this had previously been shown to result in body weight loss, reflecting an acceptable pharmacodynamic (PD) response.^17^ On day 4, mice were euthanized using a rising concentration of CO_2_, and tissue samples were collected.

#### 2.3.2 Tissue collection

The gall bladder was removed, followed by the liver. The liver was weighed and scissor-minced in ice-cold KCl (1.15% w/v) for subsequent liver subcellular fractionation. When duodenum tissue samples (approximately 10 cm of small intestine section proximal to the stomach) were taken, they were flushed with ice-cold PBS containing a protease inhibitor cocktail (Roche Diagnostics), transferred into separate tubes, flash frozen immediately in liquid nitrogen, and stored at –70 °C prior to the preparation of microsomes.

#### 2.3.3 Preparation of microsomes

Fresh liver samples were homogenized in ice-cold SET buffer (0.25 M sucrose, 5 mM EDTA, and 20 mM Tris-HCl, pH 7.4) to make a 10% (w/v) homogenate (9 mL SET buffer/1 g liver) using a Polytron PT-MR-2100 homogenizer. Liver homogenates were centrifuged at 2000 rpm (Sorvall RTH-250 rotor) for 10 minutes at 4 °C. The supernatant was then centrifuged at 15000 rpm (Beckman JA-25.50 rotor) for 20 minutes at 4 °C. The resulting supernatant was centrifuged at 29130 rpm (Sorvall TFT-45.6 rotor) for 90 minutes at 4 °C, and the microsomal pellets were resuspended in ice-cold SET buffer and stored at –70 °C.

Frozen individual duodenum sections were homogenized in SET buffer (9 mL SET buffer/1 section of duodenum) containing protease cocktail inhibitor (Roche) and phenylmethylsulfonyl fluoride (1 mM) using a Polytron homogenizer. Tissue homogenates were subjected to subcellular fractionation by differential centrifugation as described for the preparation of liver microsomes. Microsomal fractions were stored at –70 °C.

### 2.4 In vivo PK experiments

#### 2.4.1 Preparation of the NN1177 dosing solution

Stock solution of NN1177 (1 mg/mL; 219 *μ*M) was diluted in the requisite volume of vehicle provided by Novo Nordisk to obtain 0.6 *μ*M or 0.8 *μ*M solution for 3 nmol/kg or 4 nmol/kg doses, respectively.

#### 2.4.2 Preparation of the Cooperstown cocktail

The stock solutions of *S*-warfarin (20 mg/mL), omeprazole (20 mg/mL), and dextromethorphan (250 mg/mL) in DMSO were prepared, aliquoted, and stored at –20 °C. On the day of drug cassette preparation, the aliquots were thawed, and 50 *μ*L of *S*-warfarin, 200 *μ*L of dextromethorphan, and 250 *μ*L of omeprazole were mixed and vortexed. Two milliliter of PEG400 followed by 250 *μ*L of Tween 80 was then added and vortexed. Subsequently, 1650 *μ*L of freshly prepared solution of caffeine (3.03 mg/mL) in sodium citrate buffer (50 mM; pH 4.8) was added and vortexed. Finally, 600 *μ*L of midazolam (5 mg/mL injection) was added, and the mixture was vortexed and administered to 8HUMs within 1 hour after preparation.

#### 2.4.3 Preparation of phenobarbital

Sodium salt of phenobarbital was weighed out and dissolved in the corresponding volume of PBS to obtain a 4 mg/mL solution of phenobarbital.

#### 2.4.4 Preparation of substrate cassette for intravenous administration

Stock solutions of midazolam (5mg/mL; 72 *μ*L) and caffeine (3.03 mg/mL in citrate buffer [50 mM; pH 4.8]; 198 *μ*L) were added to 5730 *μ*L of dextromethorphan hydrobromide monohydrate solution in water (1.047 mg/mL). The mixture was vortexed and administered to animals within 1 hour after preparation. All formulations of the compounds were prepared on the day of administration and given at a volume of 5 mL/kg.

#### 2.4.5 PK of Cooperstown cocktail (caffeine, dextromethorphan, midazolam, omeprazole, and S-warfarin) administered orally to vehicle- or NN1177-treated 8HUM mice

8HUM mice received single daily doses of either vehicle (s.c.; 5 mL/kg) or NN1177 (s.c. at 4 nmol/kg) on days 1 to 3. When the effect of NN1177 on P450 induction was studied, all mice received the corresponding inducer (3 per oral [PO] single daily doses of SJW extract [312 mg/kg; prepared as described previously] or phenobarbital [20 mg/kg]^15^) concomitantly with NN1177 or vehicle. On day 4, all mice were administered (PO) drug cocktail (caffeine [5 mg/kg], dextromethorphan [50 mg/kg], midazolam [3 mg/kg], omeprazole [5 mg/kg], and *S*-warfarin [1 mg/kg]) and 10 *μ*L blood samples were collected from a tail vein incision at 10 minutes, 20 minutes, 40 minutes, 1 hour, 2 hours, 4 hours, 6 hours, 8 hours, 12 hours, and 24 hours after administration. The samples were then mixed with 90 *μ*L of deionized water and stored at –20 °C. Before each sample was taken, the mouse’s tail was briefly immersed in warm water to facilitate sample collection and dried with a tissue to remove any residual blood from the previous sample. On day 5, the mice were euthanized using a rising concentration of CO_2_, and the tissues were collected. In order to determine P450 expression at the start of PK profiling, satellite experimental groups were included in both SJW and phenobarbital induction studies. The treatment with inducer and vehicle or NN1177 in these groups was identical to that of animals used for the PK profiling, except on day 4, mice were euthanized using a rising concentration of CO_2_, and the tissue samples were collected and processed as described above.

#### 2.4.6 PK of caffeine, dextromethorphan, and midazolam administered intravenously as a drug cassette to vehicle- or NN1177-treated 8HUM mice

On days 1 to 3, mice received single daily doses (subcutaneously) of either vehicle (5 mL/kg) or NN1177 (4 nmol/kg). On day 4, a drug cassette (caffeine [0.5 mg/kg], dextromethorphan [3.664 mg/kg], and midazolam [0.3 mg/kg]) was injected (intravenously) into the tail vein. Ten microliter blood samples were subsequently collected from the tail vein at 10 minutes, 20 minutes, 40 minutes, 1 hour, 2 hours, 4 hours, 6 hours, 8 hours, and 24 hours after the administration, mixed with 90 *μ*L of deionized water, and stored at –20 °C. On day 5, the mice were euthanized using a rising concentration of CO_2_.

#### 2.4.7 Data analysis

PK parameters were calculated using the Plasma (200-202) model type of noncompartmental analysis module of Phoenix WinNonlin version 8.4 (Certara).

### 2.5 Biochemical measurements

#### 2.5.1 Total protein determination

The protein concentration in the microsomal fractions was determined using a modification of the method of Lowry^18^ or bicinchoninic acid^19^ and bovine serum albumin standards.

#### 2.5.2 Immunoblotting

The expression of CYP1A2, Cyp2b10, CYP2C9, CYP2D6, and CYP3A4 in microsomal samples was determined by immunoblot analysis using the following primary antibodies^15^^,^^20^: rabbit polyclonal raised against recombinant rat CYP1A2, rabbit polyclonal raised against rat CYP2B10, mouse monoclonal raised against rat CYP2C6, sheep polyclonal raised against human CYP2D6, and sheep polyclonal raised against human CYP3A4. Loading control, GRP78, was determined using rabbit polyclonal antibodies. Each loaded sample contained 10 *μ*g of total microsomal protein per lane. The positive standards were membrane preparations expressing recombinant human CYP1A2 (0.25 pmol/lane), CYP2C9 (0.25 pmol/lane), CYP2D6 (0.01 pmol/lane), and CYP3A4 (0.25 pmol/lane) or liver microsomes from phenobarbital-treated mice for Cyp2b10 (10 *μ*g total protein/lane). Human liver or intestinal microsomes were used for comparison and loaded at 10 *μ*g total protein/lane. The Western blots were visualized using an Odyssey CLx fluorescent imager (LICORbio). The digitized signal intensities were used to calculate levels of cytochromes P450 relative to GRP78. The relative expression of cytochromes P450 was normalized to the mean of that in the vehicle-treated group.

#### 2.5.3 Blood sample preparation for LC–MS/MS analysis

Blood samples were extracted and prepared using the protein precipitation method. Briefly, a volume of acetonitrile containing 20 ng/mL doneperezil (internal standard [IS]) (90 *μ*L) was added to all study samples (30 *μ*L). A volume of acetonitrile containing 20 ng/mL IS (300 *μ*L) was added to all calibration, quality controls (QCs), and zero calibrators (100 *μ*L). A volume of acetonitrile alone (300 *μ*L) was added to the blank (100 *μ*L). All resultant samples were vortexed and centrifuged for 10 minutes at 14800 rpm, 16163*g* (SIGMA SciQuip 1-14) to separate any precipitated proteins. The resultant supernatant from calibration samples, QCs, blanks, and double blanks (200 *μ*L) was then transferred to high-pressure liquid chromatography glass vials containing Milli-Q water (100 *μ*L). The resultant supernatant from the study samples (80 *μ*L) was transferred to high-pressure liquid chromatography glass vials containing Milli-Q water (40 *μ*L).

#### 2.5.4 LC–MS/MS analysis

The analysis was performed on a Waters Acquity ULPC I-class coupled to a Waters Xevo TQ-XS tandem mass spectrometer. System control, data acquisition, and data processing were performed using MassLynx (version 4.2). Chromatography was performed using an Acquity UPLC BEH C18 column, 50 × 2.1 mm, particle size 1.7 *μ*m (dextromethorphan, warfarin, and omeprazole) or a Thermo Scientific Hypersil GOLD C18 column, 50 × 2.1 mm, particle size 1.9 *μ*m (caffeine and midazolam). The column was kept at 40 °C. Mobile phase A was LC–MS grade water containing 0.01% (v/v) formic acid, and mobile phase B was acetonitrile containing 0.01% (v/v) formic acid. The gradient was run at a flow of 0.6 mL/min, starting at 95% A and 5% B. After 2.00 minutes, the gradient was changed to 5% A and 95% B and was kept stable until 2.50 minutes. At 2.60 minutes, the gradient was changed back to the starting conditions. Sample injection volume was 1 *μ*L. The MS conditions were as follows: ionization was performed in positive electrospray mode, while detection was performed in multiple reaction monitoring mode. For all analytes and ISs studied, the MassLynx QuanOptimize application was used to optimize the cone voltage (CV) for the parent ion and the collision energy (CE) required to produce the daughter ion. Specific LC–MS/MS parameters for each compound were as follows: dextromethorphan *m/z* 272.2 → 171.0, CV: 40V, CE 35V; warfarin *m/z* 309.1 → 162.9, CV: 40V, CE: 15V; omeprazole *m/z* 346.1 → 198.0, CV: 12V, CE: 10V; caffeine *m/z* 195.0 → 137.9, CV: 50V, CE: 16V; midazolam *m/z* 325.9 → 188.7, CV: 80V, CE: 26V.

#### 2.5.5 Preparation of authentic standard solutions

Caffeine, midazolam, dextromethorphan, warfarin, omeprazole, and IS stock solutions were prepared separately in DMSO to obtain a concentration of 1 mg/mL free base. The analyte(s) working solutions were prepared in acetonitrile: water (50:50 v/v) spanning the range of 2 ng/mL to 40 *μ*g/mL. The serial calibration concentration(s) of the analyte(s) were prepared in control mouse blood at a concentration range of 0.1 to 2000 ng/mL. The QC samples were prepared at a concentration of 4 ng/mL, 20 ng/mL, 400 ng/mL, and 2000 ng/mL. The concentration of IS in all samples was 20 ng/mL.

#### 2.5.6 Data processing

Sample concentrations were determined via either a linear or quadratic regression (weighting: 1/x^2^ or 1/x) on the calibration samples using the Waters TargetLynx application. Choice of regression fit and weighting were compound-specific. The correlation coefficients of all the calibration curves were ≥0.98. Nonzero calibrators were ±20% of nominal (theoretical) concentrations, and data points that failed to meet this acceptance criterion were excluded. The lower limit of quantification was determined as the analyte response with ≥3 times the analyte response of the blanks (blood with IS but not analyte). QC samples were injected throughout the analytical run, with an acceptance criterion of 66% of injected samples being within ±20% of nominal (theoretical) concentrations. All blanks and double blanks (blood with no IS or analyte) were verified as free of interference at the retention times of the analytes and IS. Sample carryover was determined in a blank injection immediately following the injection of the top calibration sample. Carryover was analytically accepted when the analyte response in the blank was <1% of the analyte response in the top calibration sample.

### 2.6 Statistical analysis

PK parameters were compared between control and NN1177-treated groups using an unpaired *t* test (two-tailed *P* values).

## 3 Results

### 3.1 Effect of NN1177 on hepatic P450 expression in 8HUM mice

**Fig. 1:**
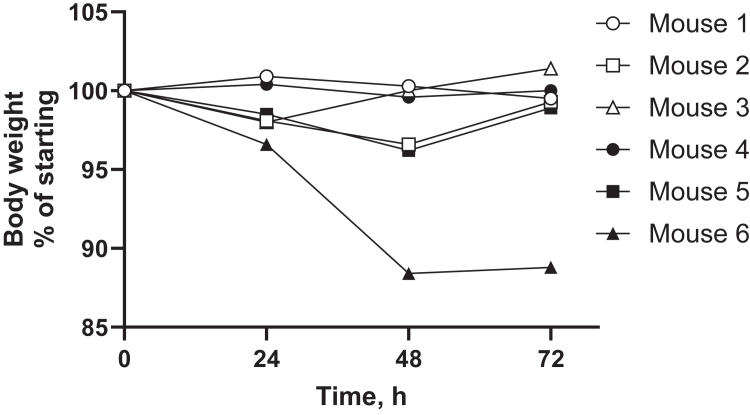
Individual body weight changes in vehicle- or NN1177-treated (3 nmol/kg) 8HUM mice. Individual body weights were measured before subcutaneous administration of vehicle (open symbols) or NN1177 (closed symbols) to 8HUM mice (3 nmol/kg; 3 doses once daily) and on day 4 before tissue collection.

**Fig. 2:**
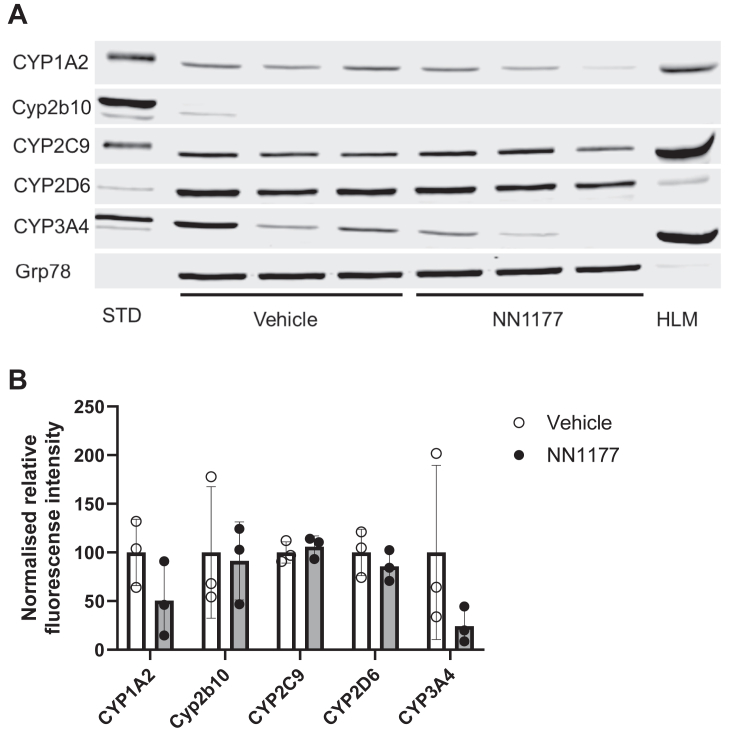
Cytochrome P450 expression in liver microsomes from vehicle- or NN1177-treated (3 nmol/kg) 8HUM mice. Hepatic microsomes were prepared from vehicle- or NN1177-treated (3 nmol/kg; s.c.; 3 doses; once daily; tissue samples collected on day 4) 8HUM mice and immunoblotted for CYP1A2, Cyp2b10, CYP2C9, CYP2D6, and CYP3A4. GRP78 was used as a loading control. The Western blots were visualized (A) using the Odyssey CLx fluorescent imager (LICORbio). The digitized signal intensities were used to calculate levels of P450s relative to GRP78. The relative expression of P450s was normalized to the mean of that in the vehicle-treated group (B). Data are mean ± SD (n = 3). Open circles/white bars or closed circles/gray bars are samples from vehicle- or NN1177-treated 8HUM mice, respectively. Standards (STD) are recombinant CYP1A2 (0.25 pmol/lane), CYP2C9 (0.25 pmol/lane), CYP2D6 (0.01 pmol/lane), and CYP3A4 (0.25 pmol/lane) or liver microsomes from phenobarbital-treated mice for Cyp2b10 (10 mg total protein/lane). HLM, human liver microsomes (10 mg total protein/lane).

**Fig. 3:**
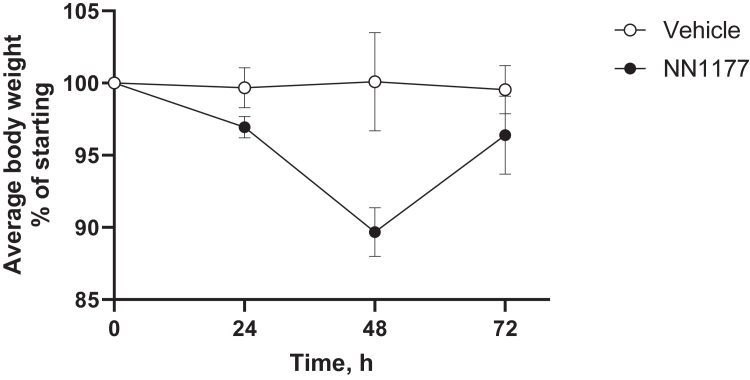
Average body weight changes in vehicle- or NN1177-treated (4 nmol/kg) 8HUM mice. Body weights were measured before subcutaneous administration of vehicle (open circles) or NN1177 (closed circles) to 8HUM mice (4 nmol/kg; 3 doses; once daily) and on day 4 before tissue collection. Data are mean ± SD (n = 3).

**Fig. 4:**
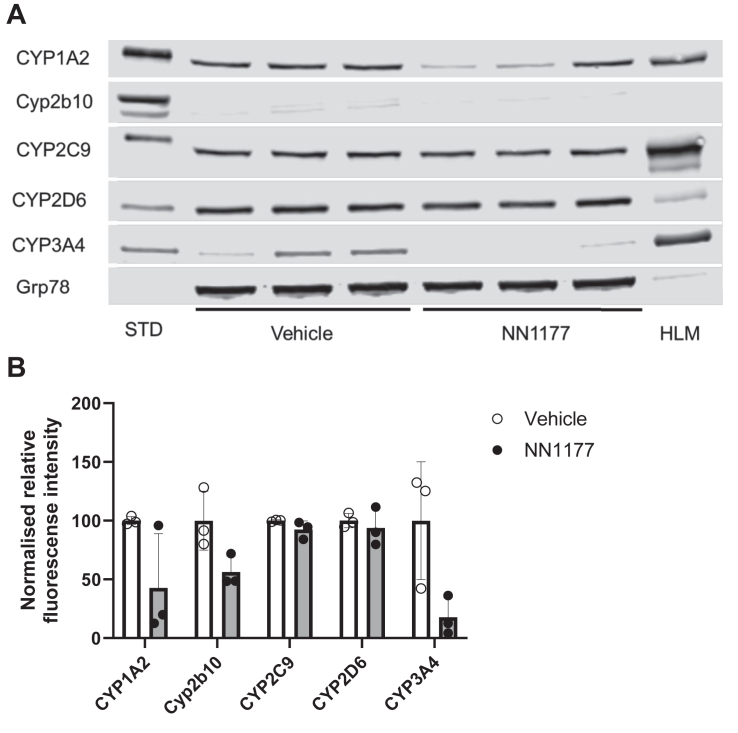
Cytochrome P450 expression in liver microsomes from vehicle- or NN1177-treated (4 nmol/kg) 8HUM mice. Hepatic microsomes were prepared from vehicle- or NN1177-treated (4 nmol/kg; s.c.; 3 doses once daily; tissue samples collected on day 4) 8HUM mice and immunoblotted for CYP1A2, Cyp2b10, CYP2C9, CYP2D6, and CYP3A4. GRP78 was used as a loading control. The Western blots were visualized (A) using the Odyssey CLx fluorescent imager (LICORbio). The digitized signal intensities were used to calculate levels of cytochromes P450 relative to GRP78. The relative expression of cytochromes P450 was normalized to the mean of that in the vehicle-treated group (B). Data are mean ± SD (n = 3). Open circles/white bars or closed circles/gray bars are samples from vehicle- or NN1177-treated 8HUM mice, respectively. Standards (STD) are recombinant CYP1A2 (0.25 pmol/lane), CYP2C9 (0.25 pmol/lane), CYP2D6 (0.01 pmol/lane), and CYP3A4 (0.25 pmol/lane) or liver microsomes from phenobarbital-treated mice for Cyp2b10 (10 mg total protein/lane). HLM, human liver microsomes (10 mg total protein/lane).

Incubation of freshly isolated primary human hepatocytes with NN1177 reduced mRNA and activities of CYP3A4 and CYP1A2. However, a subsequent clinical DDI study using the Cooperstown 5 + 1 cocktail did not reflect the effect of NN1177 observed in the preclinical study.^4^ To establish whether NN1177 affected human P450 expression in vivo in the 8HUM model, a pilot study was carried out where mice were administered 3 nmol/kg NN1177 (once daily; s.c.) for 3 days. The animal body weight was measured throughout the study, and the expression of hepatic CYP1A2, CYP2C9, CYP2D6, CYP3A4, and mouse Cyp2b10 was determined by Western blotting on day 4 (Figs. 1 and 2). A notable reduction in the expression of CYP1A2 (49%) and CYP3A4 (76%) was observed, whereas that of CYP2C9 and CYP2D6 was unaffected. Although only 1 out of 6 mice had a body weight reduction of >10%, the reduction in the P450 levels in general paralleled the reduction in body weight. In light of the marginal reduction in body weight, we increased the dose of NN1177 to 4 nmol/kg for 3 days (once daily; s.c.). Mice were euthanized on day 4. This resulted in a consistent body weight reduction of 10% at 48 hours (Fig. 3). Interestingly, body weight returned toward normal after 72 hours despite the continued administration of NN1177. Consistent with the results obtained at the lower dose, NN1177 caused a marked decrease of 58% and 82% in the expression of CYP1A2 and CYP3A4, respectively (Fig. 4, A and B). The expression of CYP2D6 and CYP2C9 was again unaffected (Fig. 4, A and B) (the suppression of CYP3A4 and CYP1A2 by NN1177 was consistent with previously published in vitro data obtained in primary human hepatocytes^4^^,^^5^).

### 3.2 Effect of NN1177 on probe drug PK in 8HUM mice

**Fig. 5:**
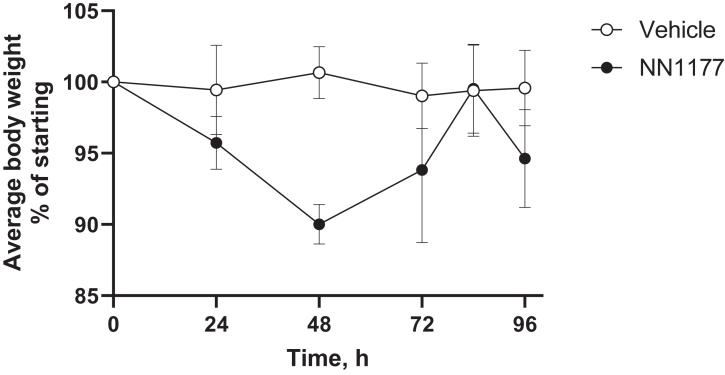
Average body weight changes during treatment of 8HUM mice with vehicle or NN1177 (4 nmol/kg) with subsequent PK profiling. Body weights were measured before subcutaneous administration of vehicle (open circles) or NN1177 (closed circles) to 8HUM mice (4 nmol/kg; 3 doses; once daily), on day 4 during PK profiling, and on day 5 before tissue collection. Data are mean ± SD (n = 3).

**Fig. 6:**
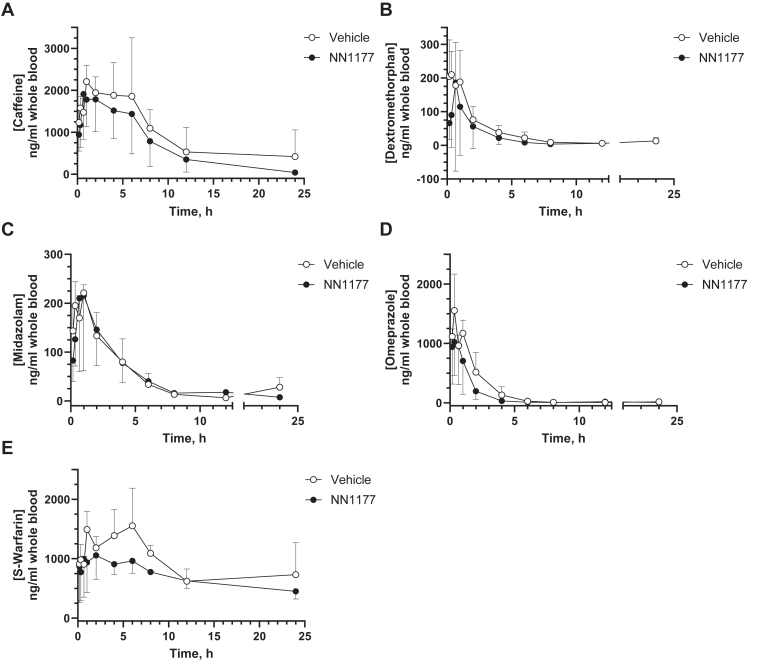
Pharmacokinetics of cytochrome P450 substrates caffeine (A), dextromethorphan (B), midazolam (C), omeprazole (D), and *S*-warfarin (E) in vehicle- or NN1177-treated (4 nmol/kg) 8HUM mice. Vehicle (open circles) or NN1177 (closed circles; 4 nmol/kg; s.c.; 3 doses; once daily) treated 8HUM mice received the drug cassette (caffeine 5 mg/kg; dextromethorphan 50 mg/kg; midazolam 3 mg/kg; omeprazole 5 mg/kg; and *S*-warfarin 1 mg/kg; PO) on day 4 and serial samples of whole blood were collected subsequently at designated time points for PK profiling. Data are mean ± SD (for vehicle-treated mice, n = 2 for 12-hour and 24-hour dextromethorphan; 24-hour midazolam, and for 8-hour and 24-hour omeprazole; for NN1177-treated mice, n = 2 for 24-hour midazolam and 24-hour omeprazole; n = 3 for all other time points).

In order to establish whether the observed suppression of CYP3A4 and CYP1A2 was reflected in a change in the PK of P450 probe drugs, we dosed 8HUM mice once daily with NN1177 (4 nmol/kg; once daily; s.c.) for 3 days, followed by PK analysis using the Cooperstown cocktail. Consistent with previous experiments, NN1177 caused a decrease in body weight (10%) after 48 hours, with some recovery after 84 hours (Fig. 5). PK analysis carried out on day 4 did not show any statistical differences between the vehicle and NN1177 treated groups for any of the P450 substrates tested (Fig. 6).

**Fig. 7:**
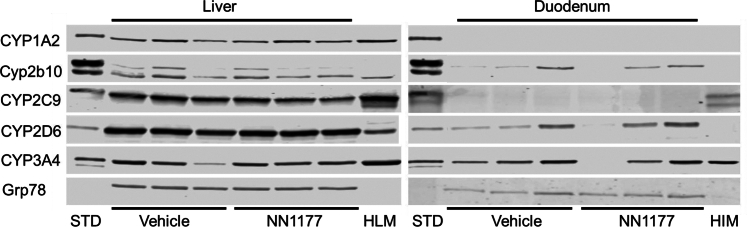
Cytochrome P450 expression in liver and duodenum from vehicle- or NN1177-treated (4 nmol/kg) 8HUM mice prepared from tissues collected after PK profiling. Hepatic and duodenum microsomes were prepared from vehicle- or NN1177-treated (4 nmol/kg; s.c.; 3 doses once daily) 8HUM mice. The tissue samples were collected following the end of PK profiling on day 5. The microsomes were immunoblotted for CYP1A2, Cyp2b10, CYP2C9, CYP2D6, and CYP3A4. GRP78 was used as a loading control. The Western blots were visualized using the Odyssey CLx fluorescent imager (LICORbio). Standards (STD) are recombinant CYP1A2 (0.25 pmol/lane), CYP2C9 (0.25 pmol/lane), CYP2D6 (0.01 pmol/lane), and CYP3A4 (0.25 pmol/lane) or liver microsomes from phenobarbital-treated mice for Cyp2b10 (10 mg total protein/lane). HIM, human intestinal microsomes (10 mg total protein/lane); HLM, human liver microsomes (10 mg total protein/lane).

It should be noted that the PK profiles (PK1) within some of the test groups exhibited variability (Table 1, Table 2, Table 3, Table 4, Table 5). Although there were differences in mean AUC_last_ C_max_, time of maximum observed drug concentration (T_max_), and half-life between vehicle and NN1177 treated groups, mean vehicle to treated ratios ranging between 4.55 for caffeine half-life and 0.42 for midazolam half-life, they were not statistically significant. It is worth noting that there was no increase in exposure for the CYP3A4 and CYP1A2 substrates. In light of the variability in PK between mice, the experiment was repeated (PK2; Supplemental Fig. 1), interindividual variability in PK profiles was thereby reduced (Table 1, Table 2, Table 3, Table 4, Table 5), and the data obtained were in overall agreement with the first experiment (PK1). The only exceptions were that statistically significant differences were observed for caffeine T_max_ (ratio 0.34) and *S*-warfarin C_max_ (ratio 0.8). The observed vehicle to treated mean AUC_last_ ratios were approximately 0.8 for both caffeine and midazolam, indicating little, if any, effect of the NN1177 treatment on the clearance of CYP1A2 and CYP3A4 specific substrates in 8HUM mice. Immunoblotting analysis of microsomal samples taken from mice on day 5 (the tissue samples were collected following the end of PK profiling) did not show any difference in P450 expression between vehicle and NN1177 treated mice (Fig. 7). The absence of a band corresponding to a P450 in duodenum sample from one of the NN1177 treated mice is likely due to proteolytic degradation in the sample, rather than the effects of NN1177 treatment.

**Table 1:** PK parameter estimates (mean ± SD) for caffeine (5 mg/kg, PO)

Inducer	PK Parameter	Treatment		Mean Ratio (Vehicle/NN1177)
		Vehicle	NN1177	
None (PK1)	AUC_last_, h×ng/mL	23000 ± 14000	16000 ± 9500	1.41
	C_max_, ng/mL	2700 ± 630	2000 ± 960	1.37
	T_max_, h	3 ± 2.9	0.78 ± 0.19	3.43
	Half-life, h	20 ± 22	3.6 ± 1.2	4.55
None (PK2)	AUC_last_, h×ng/mL	26600 ± 900	30000 ± 23000	0.80
	C_max_, ng/mL	2800 ± 300	3300 ± 690	0.84
	T_max_, h	1.2 ± 0.45	3.5 ± 1.9^a^	0.34
	Half-life, h	3.3 ± 0.23	4.4 ± 1.49	0.75
SJW	AUC_last_, h×ng/mL	9000 ± 2200	8400 ± 810	1.07
	C_max_, ng/ml	1400 ± 220	1600 ± 360	0.93
	T_max_, h	1.1 ± 0.77	3 ± 3	0.43
	Half-life, h	2.3 ± 0.76	2.5 ± 0.31	0.91
PB	AUC_last_, h×ng/mL	10000 ± 2100	15000 ± 6000	0.62
	C_max_, ng/mL	1500 ± 130	1600 ± 260	0.91
	T_max_, h	0.8 ± 0.19	4 ± 3.8	0.21
	Half-life, h	2.5 ± 0.20	3 ± 1.3	0.73

*P* < .05; unpaired *t* test; two-tailed *P* values.

a Statistically significant difference compared to vehicle-treated.

**Table 2:** PK parameter estimates (mean ± SD) for dextromethorphan (50 mg/kg, PO)

Inducer	PK Parameter	Treatment		Mean Ratio (Vehicle/NN1177)
		Vehicle	NN1177	
None (PK1)	AUC_last_, h×ng/mL	600 ± 360	300 ± 360	1.79
	C_max_, ng/mL	220 ± 89	200 ± 260	1.17
	T_max_, h	0.4 ± 0.19	0.5 ± 0.29	0.89
	Half-life, h	5 ± 2.3	3 ± 1.7	1.42
None (PK2)	AUC_last_, h×ng/mL	400 ± 250	1000 ± 860	0.25
	C_max_, ng/mL	110 ± 62	210 ± 84	0.54
	T_max_, h	0.3 ± 0.22	0.5 ± 0.25	0.65
	Half-life, h	3.2 ± 0.48	4.3 ± 1.6	0.74
SJW	AUC_last_, h×ng/mL	340 ± 54	360 ± 121	0.93
	C_max_, ng/mL	110 ± 73	170 ± 112	0.65
	T_max_, h	0.3 ± 0.10	0.2 ± 0.10	1.25
	Half-life, h	9 ± 3.7	8 ± 9.7	1.09
PB	AUC_last_, h×ng/mL	330 ± 122	540 ± 103	0.62
	C_max_, ng/mL	200 ± 99	150 ± 79	1.31
	T_max_, h	0.17 ± 0.00	0.3 ± 0.29	0.50
	Half-life, h	5.2 ± 1.22	6 ± 2.7	0.83

**Table 3:** PK parameter estimates (mean ± SD) for midazolam (3 mg/kg, PO)

Inducer	PK Parameter	Treatment		Mean Ratio (Vehicle/NN1177)
		Vehicle	NN1177	
None (PK1)	AUC_last_, h×ng/mL	900 ± 350	900 ± 490	1.00
	C_max_, ng/mL	229 ± 13	223 ± 148	1.03
	T_max_, h	0.7 ± 0.33	0.89 ± 0.19	0.75
	Half-life, h	2.1 ± 0.29	5.0 ± 1.5	0.42
None (PK2)	AUC_last_, h×ng/mL	760 ± 100	900 ± 420	0.84
	C_max_, ng/mL	220 ± 38	220 ± 70	0.99
	T_max_, h	0.33 ± 0.00	0.7 ± 0.39	0.47
	Half-life, h	5.3 ± 1.8	3.9 ± 0.88	1.34
SJW	AUC_last_, h×ng/mL	180 ± 71	130 ± 38	1.36
	C_max_, ng/mL	60 ± 28	70 ± 29	0.90
	T_max_, h	3 ± 4.5	0.28 ± 0.10	10.20
	Half-life, h	10 ± 6.6	5 ± 4.8	2.02
PB	AUC_last_, h×ng/mL	300 ± 89	490 ± 46^a^	0.61
	C_max_, ng/mL	110 ± 67	130 ± 57	0.84
	T_max_, h	0.22 ± 0.10	0.44 ± 0.19	0.50
	Half-life, h	2.9 ± 0.33	3.4 ± 1.1	0.84

*P* < .05; unpaired *t* test; two-tailed *P* values.

a Statistically significant difference compared to vehicle-treated.

**Table 4:** PK parameter estimates (mean ± SD) for omeprazole (5 mg/kg, PO)

Inducer	PK Parameter	Treatment		Mean Ratio (Vehicle/NN1177)
		Vehicle	NN1177	
None (PK1)	AUC_last_, h×ng/mL	2900 ± 830	1700 ± 1020	1.70
	C_max_, ng/mL	1700 ± 400	1000 ± 560	1.62
	T_max_, h	0.3 ± 0.10	0.4 ± 0.25	0.71
	Half-life, h	2.7 ± 1.2	3.2 ± 0.90	0.83
None (PK2)	AUC_last_, h×ng/mL	2600 ± 460	3300 ± 1000	0.78
	C_max_, ng/mL	1500 ± 340	1400 ±760	1.05
	T_max_, h	0.33 ± 0.00	0.5 ± 0.33	0.67
	Half-life, h	3.1 ± 0.18	4 ± 2.4	0.71
SJW	AUC_last_, h×ng/mL	370 ± 53	360 ± 160	1.03
	C_max_, ng/mL	280 ± 190	320 ± 200	0.88
	T_max_, h	0.28 ± 0.10	0.28 ± 0.10	1.00
	Half-life, h	10 ± 8.3	1.9 ± 0.24	5.41
PB	AUC_last_, h×ng/mL	800 ± 300	1070 ± 180	0.73
	C_max_, ng/mL	500 ± 290	580 ± 130	0.89
	T_max_, h	0.22 ± 0.10	0.28 ± 0.10	0.80
	Half-life, h	2.6 ± 0.59	3.5 ± 1.9	0.74

**Table 5:** PK parameter estimates (mean ± SD) for *S*-warfarin (1 mg/kg, PO)

Inducer	PK Parameter	Treatment		Mean Ratio (Vehicle/NN1177)
		Vehicle	NN1177	
None (PK1)	AUC_last_, h×ng/mL	22000 ± 6600	17000 ± 3700	1.32
	C_max_, ng/mL	1800 ± 390	1200 ± 430	1.48
	T_max_, h	4 ± 2.5	3 ± 2.8	1.27
	Half-life, h	30 ± 29	24 ± 11	1.20
None (PK2)	AUC_last_, h×ng/mL	30000 ± 5700	36000 ± 7500	0.83
	C_max_, ng/mL	1900 ± 330	2400 ± 250^a^	0.80
	T_max_, h	5.2 ± 1.1	5 ± 3.0	1.16
	Half-life, h	8.1 ± 1.2	8 ± 3.7	0.98
SJW	AUClast, h×ng/mL	21000 ± 2400	16000 ± 4800	1.30
	C_max_, ng/mL	1700 ± 160	1500 ± 410	1.13
	T_max_, h	4.7 ± 1.2	3 ± 3	1.71
	Half-life, h	5 ± 0.55	5.2 ± 0.33	0.96
PB	AUC_last_, h×ng/mL	19000 ± 5100	22000 ± 8500	0.85
	C_max_, ng/mL	1300 ± 420	1600 ± 570	0.86
	T_max_, h	6 ± 2.0	7 ± 5.0	0.90
	Half-life, h	5.7 ± 1.1	6.1 ± 0.49	0.94

*P* < .05; unpaired *t* test; two-tailed *P* values.

a Statistically significant difference compared to vehicle-treated.

### 3.3 Effect of NN1177 on probe drug PK following intravenous administration in 8HUM mice

**Fig. 8:**
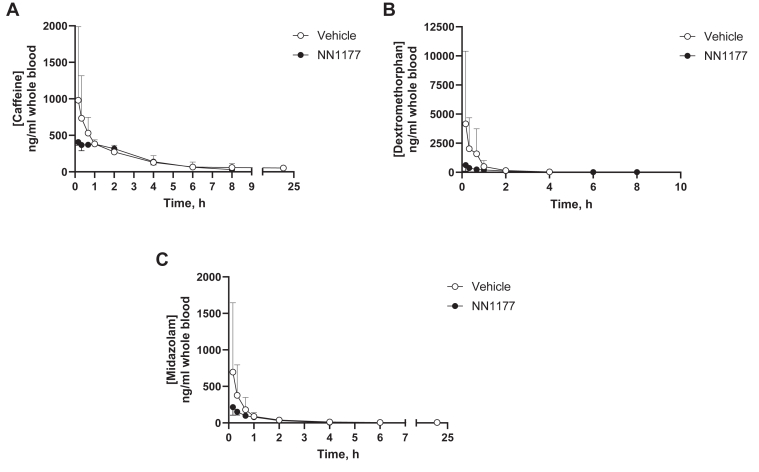
Pharmacokinetics of cytochrome P450 substrates caffeine (A), dextromethorphan (B) and midazolam (C) in vehicle- or NN1177-treated (4 nmol/kg) 8HUM mice following intravenous administration. Vehicle (open circles) or NN1177 (closed circles; 4 nmol/kg; s.c.; 3 doses; once daily) treated 8HUM mice received the drug cassette (caffeine 0.5 mg/kg; dextromethorphan 3.664 mg/kg; and midazolam 0.3 mg/kg; i.v.) on day 4 and serial samples of whole blood were collected subsequently at designated time points for PK profiling. Data are mean ± SD (for vehicle-treated group n = 2 for 8-hour caffeine and 4-hour midazolam; for NN1177-treated group, n = 2 for 6-hour and 8-hour dextromethorphan and 4-hour midazolam; n = 3 for all other time points except 24-hour caffeine and 6-hour and 24-hour midazolam for vehicle and 24-hour midazolam for NN1177 treated mice, where only one concentration value was available per group, and thus SD was not calculated).

The above data showed that despite reductions in hepatic CYP3A4 and CYP1A2 levels at early time points when livers were harvested at day 3, there was no change in the PK of the probe drugs, midazolam or caffeine. One explanation is that NN1177 was inducing other physiological and/or pharmacological changes, such as gastric emptying, which altered the bioavailability of probe substrates. To investigate this possibility, we carried out an intravenous PK study using caffeine, dextromethorphan, and midazolam. 8HUM mice were pretreated with 3 daily doses of vehicle or 4 nmol/kg NN1177 (Fig. 8). The PK profiles for individual mice were again subject to some variability (Table 6). However, there was no significant difference between vehicle- and NN1177-treated 8HUM mice for AUC_inf_, clearance, volume of distribution at steady state, or mean residence time, except for the clearance of caffeine, which was slightly higher (vehicle to treated ratio, 0.96) in the NN1177 group. It is worth noting that drug exposure was not increased for any of the probe substrates following NN1177 treatment.

**Table 6:** Intravenous PK parameter estimates (mean ± SD)

Substrate (Dose) *mg/kg*	PK Parameter	Treatment		Mean Ratio (Vehicle/NN1177)
		Vehicle	NN1177	
Caffeine (0.5)	AUC_inf_, h×ng/mL	2000 ± 1100	1600 ± 160	1.37
	CL, mL/(h×kg)	300 ± 180	320 ± 33^a^	0.91
	Vss, mL/kg	700 ± 390	870 ± 60	0.86
	MRT, h	3 ± 2.2	2.7 ± 0.33	1.23
Dextromethorphan (3.664)	AUC_inf_, h×ng/mL	3000 ± 4100	800 ± 400	4.01
	CL, mL/(h×kg)	4000 ± 2800	5000 ± 2300	0.64
	Vss, mL/kg	4000 ± 4200	9000 ± 4400	0.50
	MRT, h	1 ± 0.50	1.5 ± 0.36	0.63
Midazolam (0.3)	AUC_inf_, h×ng/mL	500 ± 500	240 ± 71	2.29
	CL, mL/(h×kg)	900 ± 660	1300 ± 340	0.69
	Vss, mL/kg	2000 ± 2300	1300 ± 520	1.54
	MRT, h	2 ± 2.7	1.0 ± 0.16	2.21

MRT, mean residence time.

*P* < .05; unpaired *t* test; two-tailed *P* values.

a Statistically significant difference compared to vehicle-treated.

### 3.4 Effect of NN1177 on P450 induction in 8HUM mice

**Fig. 9:**
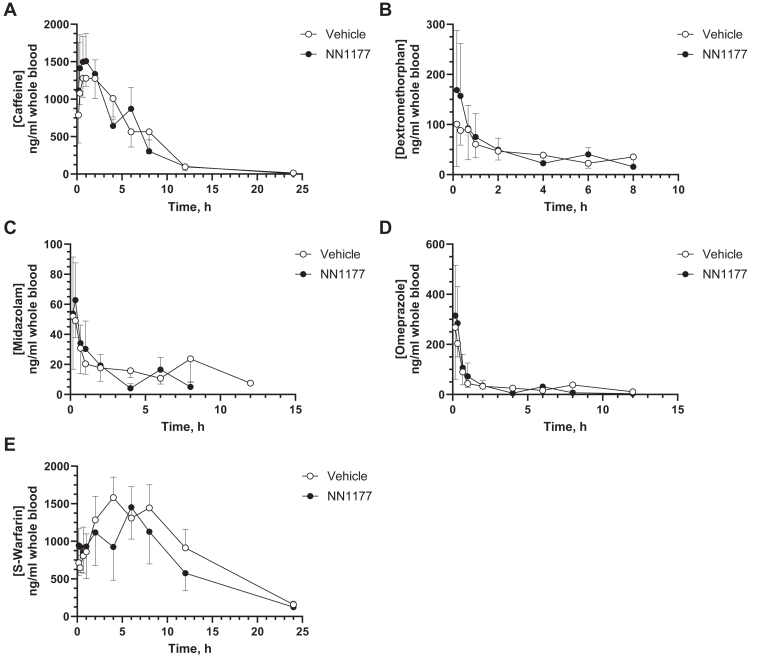
Pharmacokinetics of cytochrome P450 substrates caffeine (A), dextromethorphan (B), midazolam (C), omeprazole (D), and *S*-warfarin (E) in 8HUM mice treated concomitantly with SJW and vehicle or NN1177 (4 nmol/kg). 8HUM mice received vehicle (open circles) or NN1177 (closed circles; 4 nmol/kg; s.c.; 3 doses; once daily) concomitantly with SJW extract (312 mg/kg; PO; 3 doses; once daily) followed by administration of drug cassette (caffeine 5 mg/kg; dextromethorphan 50 mg/kg; midazolam 3 mg/kg; omeprazole 5 mg/kg; and *S*-warfarin 1 mg/kg; PO) on day 4 and subsequent collection of whole blood serial samples at designated time points for PK profiling. Data are mean ± SD (for NN1177-treated mice, n = 2 for 4-hour and 8-hour dextromethorphan; 12-hour omeprazole; n = 3 for all other time points except 24-hour caffeine, 12-hour midazolam and omeprazole for vehicle, and 24-hour caffeine for NN1177-treated mice, where only one concentration value was available per group, and thus SD was not calculated).

**Fig. 10:**
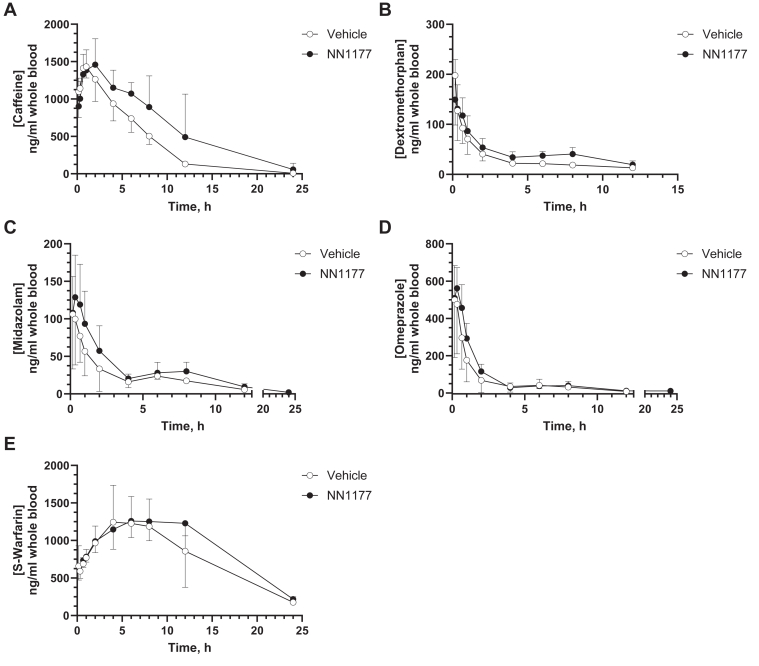
Pharmacokinetics of cytochrome P450 substrates caffeine (A), dextromethorphan (B), midazolam (C), omeprazole (D), and *S*-warfarin (E) in 8HUM mice treated concomitantly with phenobarbital and vehicle or NN1177 (4 nmol/kg). 8HUM mice received vehicle (open circles) or NN1177 (closed circles; 4 nmol/kg; s.c.; 3 doses; once daily) concomitantly with phenobarbital (20 mg/kg; PO; 3 doses; once daily) followed by administration of drug cassette (caffeine 5 mg/kg; dextromethorphan 50 mg/kg; midazolam 3 mg/kg; omeprazole 5 mg/kg; and *S*-warfarin 1 mg/kg; PO) on day 4 and subsequent collection of whole blood serial samples at designated time points for PK profiling. Data are mean ± SD (for vehicle-treated group, n = 2 for 24-hour caffeine; for NN1177-treated mice, n = 2 for 24-hour midazolam and 12-hour omeprazole; n = 3 for all other time points except 12-hour dextromethorphan and 24-hour omeprazole for vehicle- or NN1177-treated mice, respectively, where only one concentration value was available per group, and thus SD was not calculated).

The initial experiments indicated that NN1177 has the capacity to suppress constitutive P450 expression in vivo. Although the mechanism of this effect is unclear, it might be due to NN1177 affecting the transcriptional activation of CYP3A4 or CYP1A2. To test this possibility, we studied the effect of NN1177 on the induction of the P450s by the transcriptional activators, CAR and PXR. To this end, we used phenobarbital and SJW as inducing agents, respectively. 8HUM mice received 3 daily doses (PO) of the P450 inducers concomitantly with subcutaneous injections of either vehicle or 4 nmol/kg NN1177. On day 4, liver and duodenum samples were harvested from one group of animals for Western blot analysis. The remaining 2 experimental groups received the Cooperstown P450 drug cocktail, and serial whole blood samples were collected for PK analysis. Liver and duodenum tissues were also harvested at the end of the experiment (ie, day 5). NN1177 appeared to marginally reduce 8HUM body weight in the presence of either PXR or CAR activator, but not to the degree of that observed in mice not treated with an inducer (Supplemental Fig. 2, A and B). Both activators robustly induced CYP3A4 (Supplemental Fig. 3, A and B; all mice received the corresponding inducer for 3 days). However, no reduction in hepatic P450 isozyme levels was observed on NN1177 treatment. Interestingly, there appeared to be a reduction in CYPA4 and CYP2D6 levels in the gastrointestinal tract on day 5 in the phenobarbital-treated group (Supplemental Fig. 3B). This was accompanied by a reduced exposure to midazolam when compared to mice not treated with the P450-inducing agent (Table 3). No effects of NN1177 on the PK of any of the probe drugs were observed in mice treated concomitantly with either of the CAR or PXR activators (Figs. 9 and 10).

## 4 Discussion

Here, we apply the 8HUM mouse model humanized for the major P450s involved in human drug metabolism and transcription factors CAR and PXR (which regulate CYP3A4 expression) to investigate its potential for preclinical DDI prediction of the peptide NN1177, a glucagon and glucagon-like peptide 1 (GLP-1) receptor coagonist. These studies were carried out as a consequence of the discrepancy between in vitro data obtained using primary human hepatocytes as part of the preclinical assessment, which demonstrated that NN1177 suppressed the expression of CYP3A4 and CYP1A2, and a subsequent clinical trial, which showed no relevant changes in the PK of the probe drugs midazolam and caffeine.^4^ These findings were replicated in our studies here using 8HUM mice.

In addition to the study in primary human hepatocytes, the effect of NN1177 on P450 enzymes has also been investigated using more advanced in vitro systems such as HepatoPac.^5^ The results were intriguing as no relevant P450 suppression was observed, thereby reflecting the clinical observations more closely than the results from the conventional sandwich-cultured human hepatocytes. There are, however, multiple potentially confounding factors that are challenging to recapitulate in an in vitro system. Significant weight loss and/or delayed gastric emptying may, for example, impact clinical observations and subsequently lead to the observed complex DDIs. Furthermore, potential endogenous feedback mechanisms may also take place in vivo and thereby impact clinical observations. Glucagon and insulin have, for example, been shown to have opposite effects on multiple drug-metabolizing enzymes.^21^

Although the in vitro system can assess the effect of the therapeutic peptide on the P450 enzymes in the liver, the humanized mice provide the opportunity of assessing the impact on P450s in the liver coupled with the subsequent impact of the whole system on the probe substrate PK and thereby providing insight into not only the effect of the drug, but also the potential overall impact. Interestingly, the humanized mice showed that CYP3A4 can also be suppressed in the in vivo situation; however, the net effect on the probe substrate midazolam did not show any relevant changes.

Administration of NN1177 to 8HUM mice caused a reduction in bodyweight and, in agreement with the sandwich-cultured human hepatocyte data,^4^ caused a marked reduction in the expression of hepatic CYP3A4 and CYP1A2 levels. Interestingly, the change in body weight, as well as the reduction in P450 expression, was transient and returned toward normal after 4 days despite continued NN1177 administration. These data suggest that there is an adaptive metabolic response to the pharmacological activity of this hormone analog, which reverses the P450 suppression. Simonsen et al^17^ aimed to better understand the challenges associated with glucagon and GLP-1 receptor coagonists (eg, NN1177) and why these peptides often show superior efficacy preclinically, which is later not reproduced in the clinical setting. For this work, they included body weight loss, food intake, and glucose tolerance as PD markers. They showed that mice significantly improved glucose control following an acute study (single NN1177 dose of 3 or 5 nmol/kg). However, in the long-term study (drug induced obese mice received subcutaneous injections once daily for 4–5 weeks), there was a difference in the PD response depending on the time of evaluation (C_min_ or C_max_). These results led the authors to discuss the possibility of a changed receptor expression following long-term exposure compared to acute studies. Agonist-mediated receptor endocytosis and receptor desensitization may take place over time as described for G-protein–coupled receptors. These results are interesting in the context of the presented data in humanized mice, together with the previous observations in the clinic and in different types of in vitro systems. CYP3A4 expression was shown to be reduced in a dose-dependent manner in sandwich-cultured human hepatocytes following exposure for 3 days. The current study found that NN1177 also suppressed CYP3A4 in the humanized mice when the tissue samples were collected on day 4. Interestingly, if the tissue samples were harvested 1 day later (on day 5), the suppression effect on CYP3A4 was diminished. The PD effects followed the same trend in this study, where the most substantial body weight loss (10%) was observed at day 2, with a tendency to return toward baseline (effect on P450 expression was not assessed on day 2). Glucagon receptors are expressed in the liver,^17^ and glucagon has previously been shown to be involved in, for example, the regulation of CYP2C11 in rat hepatocytes.^22^ The GLP-1 receptor, on the other hand, is not expressed in the liver.^17^ The effect of NN1177 on CYP3A4 is therefore believed to be driven via glucagon receptor–mediated intracellular cascade events. If the glucagon receptor expression is changed over time, as proposed by Simonsen et al,^17^ this could explain the difference in effect on CYP3A4 depending on the time of sampling. This would also be in line with the observations from the clinical DDI trial, where a dose escalation regimen was applied to avoid tolerability issues.^4^ The study participants received an increased dose every 2 weeks for approximately 8 weeks before reaching the target dose of 4.2 mg. The target dose was then administered for 3 weeks before giving the Cooperstown 5 + 1 cocktail. The analysis of the plasma samples showed no clinically significant change in the CYP3A4 probe substrate midazolam exposure. A feasible explanation would be that the glucagon receptor expression and sensitivity could have changed during the long exposure period, and thereby did not correlate with the observations from the 3-day study in sandwich-cultured human hepatocytes, where the sensitivity and potential changes in glucagon receptor were yet to take place.

Future studies of interest include closely assessing the potential impact of sampling times following NN1177 exposure. Glucagon receptor proteomics from study samples would aid in further understanding the potential relevance of the receptor expression.

The suppression of P450s in the isolated human hepatocyte studies used mRNA levels as a measure of change (ie, a change in transcription or mRNA stability).^4^^,^^5^ In order to establish whether the suppression of P450s in 8HUM mice was due to effects on CAR/PXR-mediated transcription, we studied the effects of NN1177 on P450 induction mediated by CAR and PXR activators. It should be noted that the expression of CYP3A4 and CYP2D6 in the 8HUM mouse model is driven by human promoters, CYP2C9 off the albumin promoter, and CYP1A2 off the murine Cyp1a2 promoter. Using either SJW (PXR) or phenobarbital (CAR) as inducers, no suppression of CYP3A4 or CYP1A2 was observed. This suggests that the induction is dominant over any suppressive effect of NN1177 and could relate to the high levels of transcriptional activation induced by the activators. It does suggest that the effects of NN1177 on constitutive P450 levels are not due to changes in mRNA stability, as such an effect would be predicted to also change induced P450 levels.

In conclusion, we demonstrate that in vivo peptide-DDI studies using 8HUM mice uncover deeper insight into better understanding previous in vitro and clinical findings related to effects on P450 enzymes by a therapeutic peptide. We show that the observed level of P450 enzyme suppression depends on study conditions, and the reduction in P450 levels appeared to resolve over time, indicating a transient effect. The presented model provides a unique opportunity to simultaneously investigate the effects on P450 expression as well as the subsequent impact on the probe drug PK. It accurately predicts the outcome of the DDI assessment in clinical studies. At the same time, the study demonstrates the potential shortcomings of current DDI evaluation methods. Thus, we conclude that the 8HUM mouse model provides a promising preclinical tool that should be further investigated for its purposes in drug development. The model application can be extended to other glucagon analogs, and indeed, to the assessment of the DDIs of different, new biological modalities.

## Conflict of interest

Charles Roland Wolf is the founder of PhaSER Biomedical, a company marketing the 8HUM model. Yury Kapelyukh, Kevin D. Read, and Alastaire Kenneth MacLeod also have affiliations with the company. Carolina Säll and Charlotte Gabel-Jensen are employees and shareholders of Novo Nordisk.
