# Supplementary material for: Application of a mouse model humanized for cytochrome P450–mediated drug metabolism to predict drug-drug interactions between a peptide and small molecule drugs
Source: Drug Metab Dispos. 2025 Sep 2;53(10):100153. doi: 10.1016/j.dmd.2025.100153 (PMC12799566; doi:10.1016/j.dmd.2025.100153)
Supplement: Supplementary Figure 1 [file mmc1.pdf]

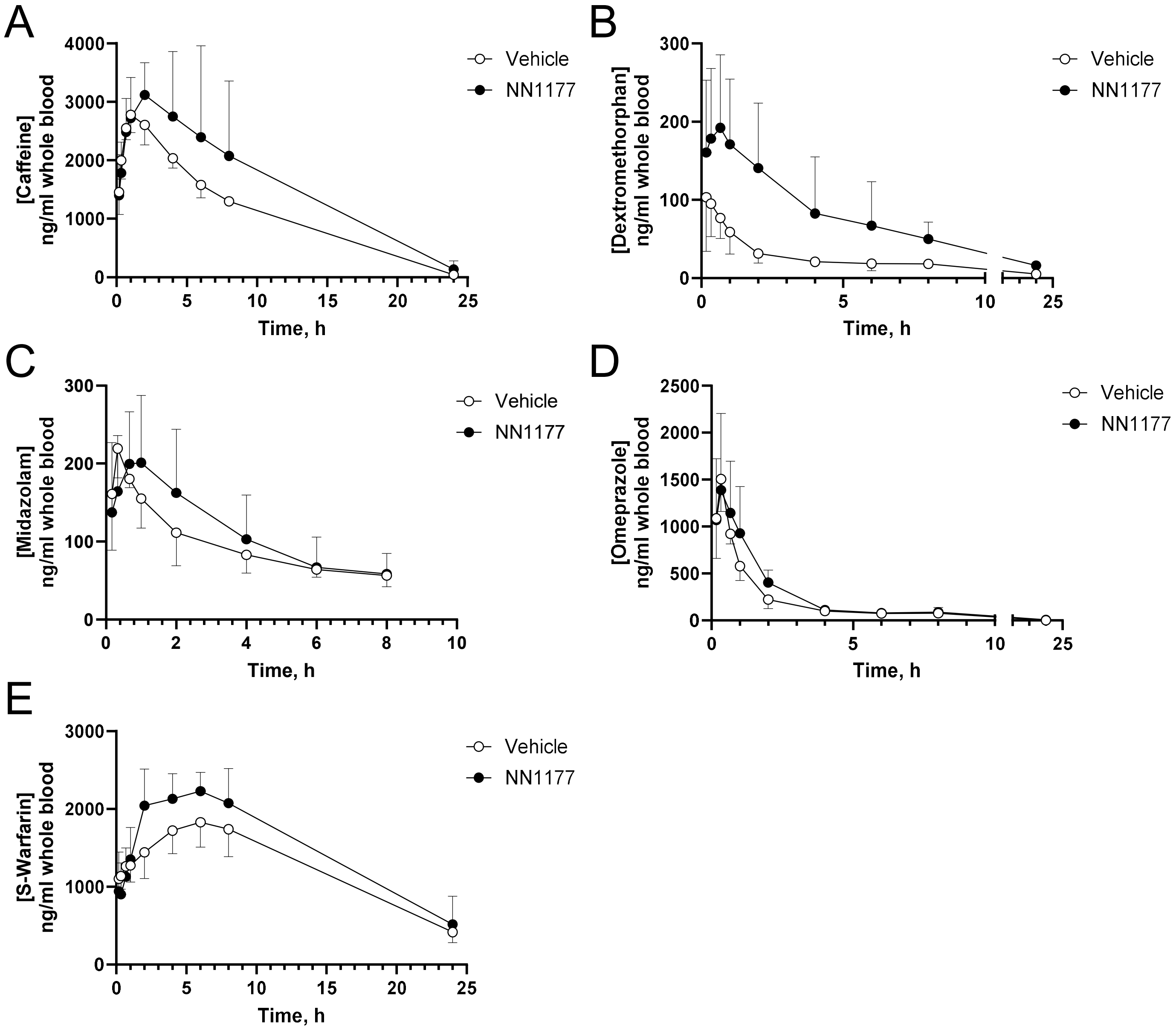

Supplemental Figure 1: Pharmacokinetics of cytochrome P450 substrates caffeine (A), dextromethorphan (B), midazolam (C), omeprazole (D), and S-warfarin (E) in vehicle or NN1177-treated (4 nmol/kg) 8HUM mice

Vehicle (open symbols) or NN1177 (closed symbols; 4 nmol/kg; subcutaneous; three doses; OD) treated 8HUM mice received the drug cassette (caffeine 5 mg/kg; dextromethorphan 50 mg/kg; midazolam 3 mg/kg; omeprazole 5 mg/kg; and S-warfarin 1 mg/kg; PO) on Day 4 and serial samples of whole blood were collected subsequently at designated time points for PK profiling. Data are mean ± SD (for NN1177 treated mice n=2 for 24 h caffeine and 24 h omeprazole; n=5 or n=4 for all other time points from vehicle or NN1177 treated mice, respectively, except 24 h dextromethorphan for both vehicle and NN1177 treated mice, where only one concentration value was available per group and thus SD was not calculated).
