# Supplementary material for: Application of a mouse model humanized for cytochrome P450–mediated drug metabolism to predict drug-drug interactions between a peptide and small molecule drugs
Source: Drug Metab Dispos. 2025 Sep 2;53(10):100153. doi: 10.1016/j.dmd.2025.100153 (PMC12799566; doi:10.1016/j.dmd.2025.100153)
Supplement: Supplementary Figure 2 [file mmc2.pdf]

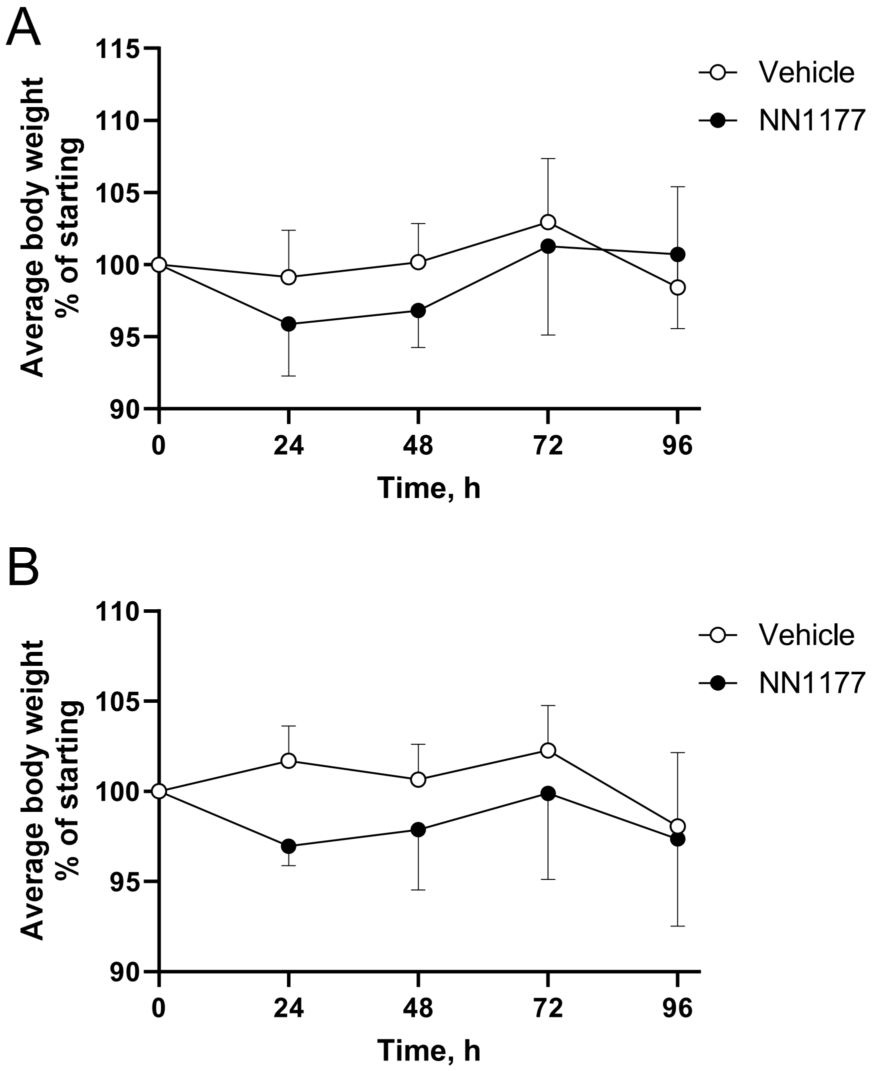

Supplemental Figure 2: Average body weight changes following treatment of 8HUM mice with cytochrome P450 inducers SJW (A) or phenobarbital (B) and concomitant administration of vehicle or NN1177 and subsequent PK profiling

Body weights were measured before subcutaneous administration of vehicle (open symbols) or NN1177 (closed symbols) to 8HUM mice (4 nmol/kg; three doses; OD), on Day 4 during PK profiling and on Day 5 before tissue collection. Concomitantly with vehicle and NN1177, mice received (PO; three doses; OD) cytochrome P450 inducers SJW (**A**; 312 mg/kg) or phenobarbital (**B**; 20 mg/kg). Data are mean ± SD (n=6 for 0h, 24h, 48h and 72h measurements for all vehicle treated groups and for 0h and 24h points for all NN-1177 treated groups; n=3 for 96h measurements for all groups; n=4 and n=5 for 48h and 72h measurements for mice treated with NN-1177 and SJW or phenobarbital respectively). Open or closed circles are body weights of vehicle or NN1177 treated 8HUM mice, respectively.
