# Supplementary material for: Application of a mouse model humanized for cytochrome P450–mediated drug metabolism to predict drug-drug interactions between a peptide and small molecule drugs
Source: Drug Metab Dispos. 2025 Sep 2;53(10):100153. doi: 10.1016/j.dmd.2025.100153 (PMC12799566; doi:10.1016/j.dmd.2025.100153)
Supplement: Supplementary Figure 3 [file mmc3.pdf]

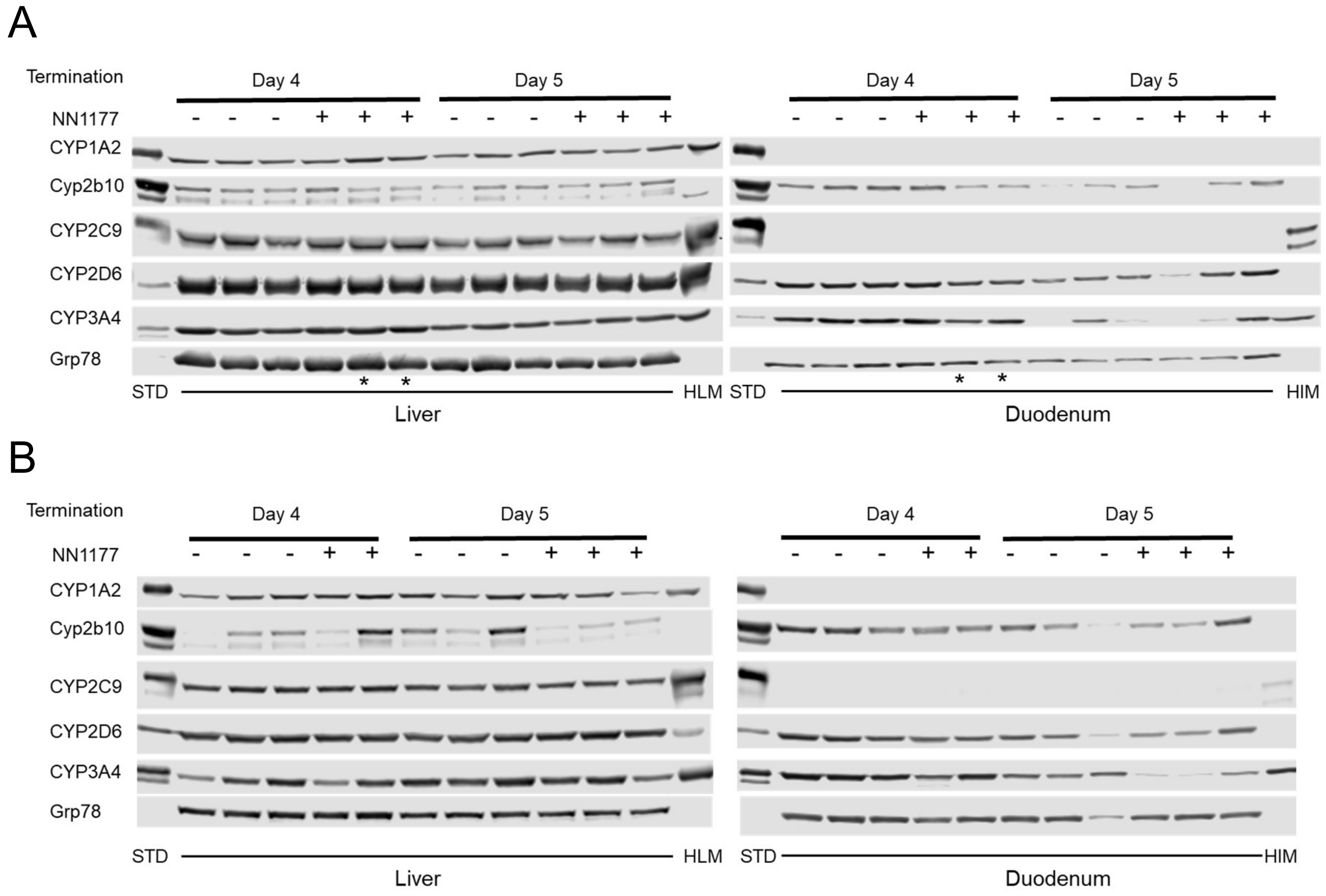

Supplemental Figure 3: Cytochrome P450 expression in liver and duodenum microsomes from vehicle or NN1177 (4 nmol/kg) treated 8HUM mice concomitantly dosed with P450 inducers SJW (A) or phenobarbital (B) and used in the subsequent PK profiling

Hepatic and duodenum microsomes were prepared from vehicle or NN1177-treated (4 nmol/kg; subcutaneous; three doses OD) 8HUM mice concomitantly dosed with SJW (**A**; 312 mg/kg; PO; three doses; OD) or phenobarbital (**B**; 20 mg/kg; PO; three doses; OD). **Day 4** are samples from satellite groups, where tissues were collected on the next day after the last dose of inducer and vehicle or NN1177 (* - mice were culled on day 2 due to animal welfare reasons). **Day 5** are samples from tissues collected after the treatment and subsequent PK profiling. The microsomes were immunoblotted for CYP1A2, Cyp2b10, CYP2C9, CYP2D6, and CYP3A4. GRP78 was used as a loading control. The Western blots were visualized using Odyssey CLx fluorescent imager (LICORbio). Standards (STD) are recombinant CYP1A2 (0.25 pmol/lane), CYP2C9 (0.25 pmol/lane), CYP2D6 (0.01 pmol/lane) and CYP3A4 (0.25 pmol/lane) or liver microsomes from phenobarbital treated mice for Cyp2b10 (10 mg total protein/lane). HLM and HIM are human liver or intestinal microsomes, respectively (10 mg total protein/lane).
